# Use of AccuVein AV500 in differentiating veins from arteries during microsurgical varicocelectomy

**DOI:** 10.1002/bco2.271

**Published:** 2023-07-03

**Authors:** Mitsuru Komeya, Tomoki Saito, Shinnosuke Kuroda, Haru Hamada, Ai Miyakoshi, Teppei Takeshima, Tomonari Hayama, Mariko Murase, Yasushi Yumura, Kazuhide Makiyama

**Affiliations:** ^1^ Department of Urology Yokohama City University Graduate School of Medicine Yokohama Japan; ^2^ Department of Urology Yokohama City University Medical Center Yokohama Japan; ^3^ Department of Obstetrics and Gynecology Yokohama City University Medical Center Yokohama Japan

**Keywords:** accuvein, male infertility, microsurgical varicocelectomy, near‐infrared light, varicocele, vascular access imaging device

A varicocele is an abnormal enlargement of the pampiniform venous plexus in the scrotum. This physical condition is present in 11.7% of men, 25.4% of men with abnormal semen analysis and 40% of men presenting with infertility.^1^ Surgical treatment by ligation of the venous plexus is performed to improve semen analysis parameters and decrease sperm DNA damage.^2^, ^3^ Although there are no recent data, a previous meta‐analysis showed that varicocelectomy might improve outcomes following assisted reproductive techniques in oligozoospermic men.^4^

The following surgical approaches may be used: scrotal operation, inguinal approach, high ligation, microsurgical inguinal or subinguinal approach, and laparoscopy. Microsurgical varicocelectomy is considered the most effective approach due to the fewer complications and lower recurrence rates associated with it, when compared with the other techniques.^5^ However, even if the microsurgical approach was chosen, severe complications remained, including iatrogenic testicular artery ligation (1%) and secondary testicular atrophy (5% in iatrogenic artery ligation).^6^ In a randomised controlled trial, the use of Doppler ultrasonography during microsurgical varicocelectomy shortened operative time and increased the number of protected arteries and ligated spermatic veins.^7^ Therefore, we adapted subinguinal microsurgical varicocelectomy using Doppler ultrasonography. However, this procedure requires microsurgical training and the use of Doppler ultrasonography to identify each blood vessel. These factors make the procedure complex and extend the operative time. In rare cases, it may also be difficult to differentiate the arteries and veins.

In this study, we aimed to evaluate the ability of AccuVein® (AV500, AccuVein Inc., Medford, New York, USA) to identify internal spermatic veins in real time during microsurgical varicocelectomy. AccuVein® is a highly portable vascular access imaging device that can support the decision of the optimal venipuncture site and avoid potential complications. Projected near‐infrared light through the skin is absorbed by reduced haemoglobin in the vein and is reflected by the surrounding tissue. Arteries with oxidised haemoglobin and lymphatic vessels without haemoglobin reflect near‐infrared light like the surrounding tissue. After the reflected light is captured and processed, the information is digitally projected onto the surface of the skin. The veins are illuminated with green lines, and other tissues including arteries and lymphatic vessels are depicted as black on the skin surface. Therefore, it provides a real time, accurate image of the patient's venous pattern. The device is also widely applied for various surgical procedures, including lymphatic venous anastomosis and trocar insertion during robot‐assisted radical prostatectomy.^8^, ^9^, ^10^

In this pilot study, 10 male infertile patients who underwent microsurgical varicocelectomy with subinguinal approach under AccuVein® visualisation between July 2020 and December 2020 in the Yokohama City University Medical Center were enrolled. Briefly, after making an incision in the skin, the spermatic cord was detected outside the external inguinal ring and pulled out of the wound. The ligation of the external spermatic veins and the incision of the external, cremasteric and internal spermatic fascia were performed. The remaining internal spermatic cord was observed to identify spermatic veins from arteries using the surgical microscope. In this microscopic procedure, near‐infrared light from AccuVein® was continuously projected on the spermatic cord. Once the blood vessels have been identified by normal visible light observation, the visible light is switched off and the blood vessels shown as green lines by AccuVein® are recorded as internal spermatic veins. Subsequently, using conventional Doppler ultrasonography, we determined whether the same vessel was a vein. Blood vessels depicted as black by AccuVein® were also assessed by Doppler ultrasonography. After identifying arteries and veins by Doppler ultrasonography, the veins were ligated, and the arteries and lymphatic vessels were preserved.

Based on the intraoperative record, the discrimination rates and times of blood vessels by AccuVein® were retrospectively compared using conventional Doppler ultrasonography. The determination of whether each vessel is venous or arterial using both methods was recorded by paramedical personnel in the operating room. The time spent identifying each vessel was also counted with a stopwatch in the operation room. All patients had left varicocele only. Among these, four had grade 2 varicocele and six had grade 3 varicocele. The mean age, right testicular volume, left testicular volume and operative duration were 33.60 ± 4.58 years, 17.50 ± 3.87 mL, 16.10 ± 4.63 mL and 96.90 ± 19.47 min, respectively. Moreover, the mean numbers of veins, arteries and lymphatic vessels in the internal spermatic fascia were 14.50 ± 5.66, 4.20 ± 1.32 and 8.20 ± 3.99, respectively. At the first follow‐up visit (2 weeks post‐surgery) and second follow‐up visit (2 months post‐surgery), there were no postoperative complications. Regarding the discrimination of blood vessels, all 145 veins confirmed by conventional methods are illustrated with green lines (Figure 1), while all 42 arteries confirmed by conventional methods are depicted as black (Figure 1). The sensitivity, specificity, positive predictive value, negative predictive value and diagnostic accuracy of AccuVein® were calculated by 2 × 2 contingency table. At 95% confidence interval (CI), the sensitivity and specificity of AccuVein® were 98.1% (95% CI 96.2%–100%) and 93.8% (95% CI 87.7%–100%), respectively. The positive and negative predictive value and diagnostic accuracy were 98.1% (95% CI 96.2%–100%), 93.8% (95% CI 87.7%–100%) and 98.5% (95% CI 97.1%–100%), respectively. The mean time for vessel identification by AccuVein® were significantly shorter than conventional methods (11.11 ± 3.65 s and 389.7 ± 141.4 s, respectively; *p* = 0.003). These preliminary data showed that AccuVein® has the potential to differentiate veins from arteries in microsurgical varicocelectomy. In addition, the conventional method of distinguishing between veins and arteries by checking the presence or absence of pulsation of blood vessels using Doppler ultrasonography is time consuming; however, this device can facilitate distinction on direct visualisation. Because identification of blood vessels is repeated 30–40 times in microscopic varicocelectomy, this device can be expected to shorten operating times. As there is no need to learn how to use Doppler ultrasonography, the device can reduce iatrogenic ligation of arteries during microscopic varicocelectomy, even by inexperienced surgeons.

**FIGURE 1:**
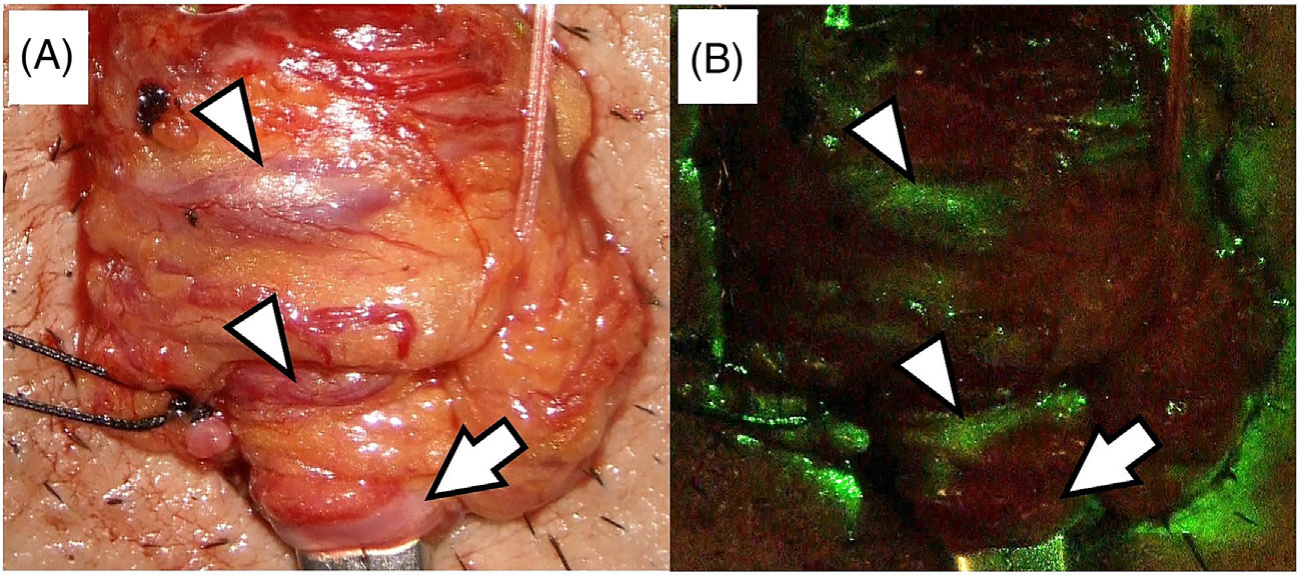
The testicular cord under microsurgical varicocelectomy. (A) The bright‐field image showed two internal spermatic veins (arrowheads) and one internal spermatic artery (arrow). (B) Veins were visualised as green (arrowheads) and arteries as black (arrow) vessels under AccuVein® visualisation.

The study limitations are as follows. First, this pilot study included a small number of patients and retrospectively analysed the discriminatory ability of the device. Second, the effect of assisted reproductive techniques on the improvement of sperm quality and outcomes was not evaluated. Third, the long‐term postoperative complications including hydrocele and testicular atrophy were also not evaluated. After validating the ability to discriminate veins in a large number of patients and further investigation by a prospective study or randomised control trials, a comparison with the Doppler ultrasonography should be executed to evaluate the surgical outcome. The study omitted the evaluation of microveins less than 1 mm in diameter because the projection of green light on the surface of the vein was weaker than that on the skin. Therefore, the projection system should be improved to evaluate the discriminatory ability of whole veins, including the microveins. The device could not directly detect arteries. In the future, we plan to optimise the device to directly identify arteries because it will enable us to preserve several internal spermatic arteries easily and immediately. The rapid and reliable preservation of arteries will contribute to reducing the operative time, preventing arterial ligation and reducing postoperative complications. This preliminary study will be the first step towards the realisation of these advances. In conclusion, this is the first report demonstrating the ability of AccuVein® to easily discriminate veins from arteries in real time during microsurgical varicocelectomy.

## AUTHOR CONTRIBUTIONS

All the authors contributed to the conception and designing of the article. Mitsuru Komeya wrote the manuscript. All the other authors did a critical review of the article. All the authors read the final manuscript and approved its submission.

## CONFLICT OF INTEREST STATEMENT

The authors declare no conflicts of interest.
